# Optical genome mapping of structural variants in Parkinson’s disease-related induced pluripotent stem cells

**DOI:** 10.1186/s12864-024-10902-1

**Published:** 2024-10-19

**Authors:** Joanne Trinh, Susen Schaake, Carolin Gabbert, Theresa Lüth, Sally A. Cowley, André Fienemann, Kristian K. Ullrich, Christine Klein, Philip Seibler

**Affiliations:** 1 Institute of Neurogenetics, University of Lübeck and University Hospital Schleswig-Holstein, Ratzeburger Allee 160, 23562 Lübeck, Germany; 2 James and Lillian Martin Centre for Stem Cell Research, Sir William Dunn School of Pathology, University of Oxford, Oxford, UK; 3 Division Scientific IT Group, Max Planck Institute for Evolutionary Biology, 24306 Plön, Germany

**Keywords:** Optical genome mapping, iPSCs, Parkinson’s disease, Structural variants

## Abstract

**Background:**

Certain structural variants (SVs) including large-scale genetic copy number variants, as well as copy number-neutral inversions and translocations may not all be resolved by chromosome karyotype studies. The identification of genetic risk factors for Parkinson’s disease (PD) has been primarily focused on the gene-disruptive single nucleotide variants. In contrast, larger SVs, which may significantly influence human phenotypes, have been largely underexplored. Optical genomic mapping (OGM) represents a novel approach that offers greater sensitivity and resolution for detecting SVs. In this study, we used induced pluripotent stem cell (iPSC) lines of patients with PD-linked *SNCA* and *PRKN* variants as a proof of concept to (i) show the detection of pathogenic SVs in PD with OGM and (ii) provide a comprehensive screening of genetic abnormalities in iPSCs.

**Results:**

OGM detected *SNCA* gene triplication and duplication in patient-derived iPSC lines, which were not identified by long-read sequencing. Additionally, various exon deletions were confirmed by OGM in the *PRKN* gene of iPSCs, of which exon 3–5 and exon 2 deletions were unable to phase with conventional multiplex-ligation-dependent probe amplification. In terms of chromosomal abnormalities in iPSCs, no gene fusions, no aneuploidy but two balanced inter-chromosomal translocations were detected in one line that were absent in the parental fibroblasts and not identified by routine single nucleotide variant karyotyping.

**Conclusions:**

In summary, OGM can detect pathogenic SVs in PD-linked genes as well as reveal genomic abnormalities for iPSCs that were not identified by other techniques, which is supportive for OGM’s future use in gene discovery and iPSC line screening.

**Supplementary Information:**

The online version contains supplementary material available at 10.1186/s12864-024-10902-1.

## Introduction

Genomic structural variants (SVs) are diverse in type and size (from ~ 50 bp to megabases of sequence) and comprise many subclasses that consist of unbalanced copy number variants (CNVs), such as deletions, duplications and insertions of genetic material, as well as balanced rearrangements, such as inversions and inter-chromosomal and intra-chromosomal translocations [1]. Identifying SVs is essential for genome interpretation but has been limited in the past due to the lack of available genome technologies. For example, the neurodegenerative disorder Parkinson’s disease (PD) has a complex etiology with genetic risk factors, environmental and lifestyle factors, and age. When including *GBA1* variants as the strongest known risk factor for PD, genetic forms of PD explain the etiology in about ~ 14% of all PD patients [2]. However, a large part of PD remains genetically unexplained, although heritability estimates show that the genetic component of PD is 27% [3]. Identification of PD-related genetic risk factors has to date been primarily limited to the study of single nucleotide variants (SNVs) suggesting that there are many more unknown genetic causes to be found. Notably, duplications and triplications in the *SNCA* gene have been reported as CNVs that cause autosomal dominant PD [4, 5]. In autosomal recessive PD, *PRKN*, *PINK1* and *PARK7* harbor whole exon deletions or multiplications, and CNVs between exon 2–5 in *PRKN* are more frequent due to a recombination hotspot [6–8]. To understand the interactions among the various genetic mechanisms implicated in the pathology of PD, patient-derived induced pluripotent stem cells (iPSCs) provide the potential to model the diseased cell type with the entire genetic background of the patient. However, iPSCs can develop genetic abnormalities during reprogramming or prolonged cell culture [9] that can interfere with the usability of iPSCs as a model system for diseases.

Optical genomic mapping (OGM) is a new method that can provide greater sensitivity and resolution of SVs [10]. OGM generates images of molecules with an average N50 > 250 kb and can generate ∼300× genome coverage per flow cell [11]. The current technology detects insertions and deletions as small as 500 bp [12], which is a much higher resolution compared to karyotyping and CNV microarrays, and it allows the detection of balanced and unbalanced events. For larger SVs and repeat expansions (REs), long-read sequencing can be useful. In recent years, the Oxford Nanopore long-read sequencing technology, as a type of third-generation sequencing, has offered an easier laboratory analysis and workflow [13, 14]. In this study, we set out to use a combination of Bionano OGM and Oxford Nanopore long-read sequencing technologies as proof-of-concept to (i) detect known pathogenic structural variants in PD and (ii) serve as a thorough genetic screening for iPSCs.

## Methods

### Patient and control demographics

Five patient and two control skin fibroblast-derived iPSC lines were examined by OGM in this study. The *SNCA* triplication iPSC line (SFC831-03-05) is from a female patient with severe PD who had an age at onset (AAO) in her late 30s, and age at biopsy (AAB) at 55 years. The *SNCA* duplication iPSC line (SFC827-03-02) originates from a female patient with PD, AAO 33 years, AAB 46 years. The iPSC line with *PRKN* compound heterozygous exon 2 and exons 3–5 deletions (iPS-L-3034) is from a male patient with PD, AAO 38 years, AAB 53 years. The *PRKN* exon 1 deletion iPSC line (iPS-L-3244) is derived from a female patient with an AAO 39 years, AAB 45 years. The *PRKN* exon 4 deletion line (iPS-L-10312) originates from a female patient, AAO 39 years, AAB 52 years. The donors of the two male control lines SFC065-03-03 and SFC163-03-01 had an AAB of 65 and 66 years, respectively.

### Fibroblasts and induced pluripotent stem cell culturing

Skin biopsies were used to establish fibroblast cultures, which were maintained in Dulbecco’s modified Eagle’s medium supplemented with 10% fetal bovine serum (Thermo Fisher Scientific) and 1% penicillin/streptomycin (Thermo Fisher Scientific).

All iPSC lines were generated by overexpression of OCT4, SOX2, KLF4, and cMYC using Sendai virus to infect the fibroblast cultures according to the manufacturer’s protocol (CytoTune Reprogramming Kit; Thermo Fisher Scientific). iPSC lines SFC831-03-05 (https://hpscreg.eu/cell-line/STBCi024-C) [15], SFC065-03-03 (https://hpscreg.eu/cell-line/STBCi057-A), SFC163-03-01 (https://hpscreg.eu/cell-line/STBCi102-A), and iPS-L-3244 [16] have been reported previously. SFC827-03-02, iPS-L-3034, and iPS-L-10,312 were characterized as part of this study (Supplementary Fig. 1). In brief, total RNA from cell pellets was isolated using the RNeasy Mini Kit (Qiagen) according to manufacturers’ instructions and reverse transcribed into cDNA (First Strand cDNA Synthesis Kit, Thermo Fisher Scientific). Quantitative real-time PCR was performed on the Lightcycler 480 (Roche) using Maxima SYBR Green (Thermo Scientific) to analyze levels of pluripotency markers OCT4, SOX2, and GDF3. For assessment of clearance of CytoTune Sendai virus-delivered reprogramming genes, the cDNA product was used in an RT-PCR reaction according to the manufacturer’s instructions, and run on an agarose gel. Positive controls (fibroblasts infected 5 days previously) were run in parallel. Primers are published elsewhere [17]. For FACS analysis of pluripotency markers Tra-1-60 and NANOG (B119983, IgM-488, Biolegend; 2985 S, IgG-647, Cell Signaling), cells were fixed in 2% paraformaldehyde, permeabilized in 100% methanol and measurement was by FACS Calibur (Becton Dickinson), with analysis using FlowJo [14]. All iPSC lines were cultured in mTeSR1 medium (StemCell Technologies) onto Matrigel-coated plates (BD Bioscience).

### Bionano optical mapping

**Fig. 1:**
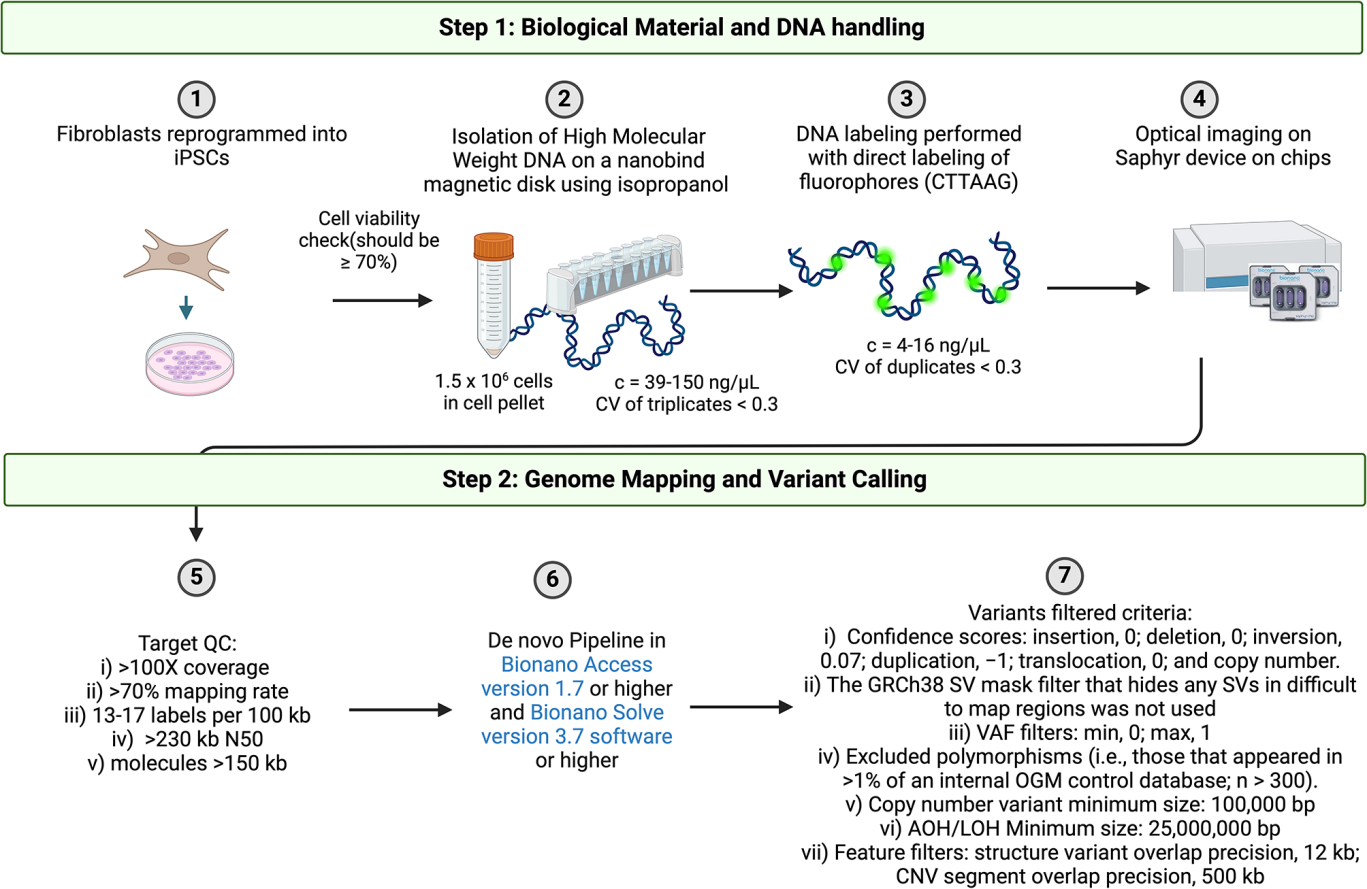
Overview of Bionano Optical Genomic Mapping Workflow for induced pluripotent stem cells (iPSCs). CV, coefficient of variation; QC, quality check; CNV, copy number variant; kb kilobase; SV, structural variant; VAF, variant allele frequency; AOH/LOH, absence or loss of heterozygosity. Created with BioRender.com

Fibroblast or iPSC-derived DNA was extracted and used for the optical mapping. Ultra-high-molecular-weight DNA was isolated, labeled, and processed for analysis on the Bionano Genomics Saphyr platform. One and a half million frozen cells were digested with Proteinase K and lysed using Lysis and Binding Buffer. DNA was precipitated on a nanobind magnetic disk using isopropanol and washed using wash buffers A and B. The ultra-high-molecular-weight DNA bound to nanobind disk was eluted and quantified using Qubit broad-range double-stranded DNA assay kits (Thermo Fisher Scientific). DNA labeling was performed using 750 ng of ultra-high-molecular-weight DNA with direct labeling (DL)-green fluorophores at a specific six-base sequence motif (CTTAAG) using Direct Labeling Enzyme 1 reactions. Following the labeling reaction, the Direct Labeling Enzyme was digested with Proteinase K, and the DL-green was removed using adsorption membranes. The DNA backbone was then stained blue and quantified using Qubit high-sensitivity double-stranded DNA assay kits. Optical imaging was performed on the Saphyr instrument by loading the fluorescently labeled DNA molecules onto the flow cells of Saphyr chips. Analytical quality control (QC) targets are set to achieve > 100X effective coverage of the genome, > 70% mapping rate, 13 to 17 label density (labels per 100 kb), and > 230 kb N50 (molecules > 150 kb). Genome analysis was performed using the optimized de-novo pipeline included in the Bionano Access version 1.7 or higher and Bionano Solve version 3.7 software for all the samples. Briefly, single molecules were used to generate the assembly of the genome, with the direct alignment of the consensus maps to GRCh38 to detect germline SVs (insertions, duplications, deletions, inversions, and translocations) based on the differences in the alignment of labels between the sample and the reference assembly. In addition, a coverage-based algorithm enabled the detection of large CNVs and aneuploidies. For data analysis, the variants were filtered using the following criteria: (i) The manufacturer’s recommended confidence scores: insertion, 0; deletion, 0; inversion, 0.07; duplication, − 1; translocation, 0; and copy number (CN), 0.99 (low stringency, filter set to 0). (ii) The GRCh38 SV mask filter that hides any SVs in difficult to map regions was excluded from analysis. (iii) To narrow the number of variants to be analyzed, we filtered out polymorphisms (i.e., those that appeared in > 1% of an internal OGM control database; *n* > 800). (iv) CNV minimum size was set to 100,000 bp. (v) Filters for absence of heterozygosity (AOH)/loss of heterozygosity (LOH) were set to a minimum size of 25,000,000 bp. Lastly, (vi) the variants were further filtered out by annotating for variants in diseases/disorders. The overlap is defined as one label overlap with a 12 kb buffer corresponding to the average label distance ± SD around each gene/loci for SVs and 500 kb for CNVs. An overview of the workflow is illustrated in Fig. 1.

### Oxford nanopore (ONT) sequencing

Whole genome sequencing was performed according to the manufacture protocol for genomic DNA using the SQK-LSK114 (ONT) library kit. 1.5 µg of the ultra-high-molecular-weight DNA isolated for Bionano Optical Mapping was treated first with T7 Endonuclease (incubation for 15 min. at 37 °C followed by AmpureXP bead clean up) or sheared in a g-TUBE (Covaris^®^) at 2500 rpm for 1 min twice with inverting the tube in between. After a DNA repair and end-prep step, the ONT provided sequencing adapter was ligated. The final product was completely loaded on an R10 flow cell on the PromethION. Base-calling was performed with Dorado version 7.2.13. Only reads with a base-calling accuracy of over 90% were included. For the alignment to the reference sequence, Minimap2 (version v2.22) was used [18]. The handling of SAM/BAM files as well as the calculation of the coverage was performed with SAMtools (version 1.15) [19]. Only reads with an alignment length over 1 kb were included in the analysis. Finally, the detection of SVs was performed using Sniffles (version 2.2) [20]. As Sniffles has reported limitations to call large (i.e., > 50 kb) deletions and CNVs, we adjusted the “--long-del-coverage” and “--long-dup-coverage” parameters within Sniffles to 1.5 and 1.1, respectively, in addition to running the software with default parameters [21]. Furthermore, we utilized NGMLR (v.0.2.7) to align the sequencing data and repeated the variant calling with Sniffles, as it has been shown to improve the variant calling of large duplications [21]. The software NanoPlot (version 1.38.0) was used to analyze the quality of the reads. All analyses were performed with the reference genome build hg38.

## Results

An average of 2,176,866 molecules were run per sample for Bionano optical mapping with a total length of 518,777 Mb across all molecules, and an average length of 241.14 kb, N50 of 233.39 kb, and a label density of 16.44/100 kb per sample, resulting in an average coverage of 167.98X (Table 1a). The Nanopore sequencing runs generated an average of 8,146,189 reads, a mean phred score of 14.5, a mean read length of 10.70 kb, N50 of 15.41 kb, and an average coverage of 29.89X (Table 1b).

**Table 1a:** Summary of molecule statistics from the Bionano Run

Sample (biomaterial)	Total number of molecules	Total length (Mb)	Average length (kb)	Molecule N50 (kb)	Label density (x/100 kb)	Coverage of the reverence (X)
SFC831-03-05 (iPSC)	1,804,468	416310.76	230.71	219.57	15.65	134.80
SFC827-03-02 (iPSC)	2,411,910	567203.28	235.17	231.34	16.79	183.66
iPS-L-3034 (iPSC)	1,216,144	312589.23	257.03	239.63	16.84	101.22
L-3034 (Fibroblast line)	1,833,639	442215.53	241.17	237.37	14.55	143.19
iPS-L-3244 (iPSC)	2,465,822	581308.75	235.75	230.89	16.61	188.23
iPS-L-10312 (iPSC)	2,057,001	613401.31	298.20	292.37	16.70	198.62
SFC065-03-03 (iPSC)	2,519,403	524517.25	208.19	200.33	16.23	169.84
SFC163-03-01 (iPSC)	2,763,315	616108.41	222.96	219.63	16.28	199.50
Average	2,176,866	518777.00	241.14	233.39	16.44	167.98

Mb = megabase; kb = kilobase

**Table 1b:** Summary of read statistics from the Oxford Nanopore Run

Sample (biomaterial)	Total number of reads	Mean read quality (Phred)	Mean read length (kb)	Read length N50 (kb)	Coverage of the reverence (X)
SFC831-03-05 (iPSC)	9,393,834	16.3	9.16	9.82	34.82
SFC827-03-02 (iPSC)	10,081,904	12.2	10.14	18.67	33.82
iPS-L-3034 (iPSC)	5,498,166	15.7	11.41	12.55	23.49
iPS-L-3244 (iPSC)	7,095,698	12.2	10.74	22.58	27.00
iPS-L-10312 (iPSC)	8,661,344	16.0	12.04	13.41	30.30
Average	8,146,189	14.5	10.70	15.41	29.89

kb = kilobase

After applying filtering steps (e.g. confidence scores, the GRCh38 SV mask filter, VAF, polymorphisms (i.e., those that appeared in > 1% of an internal OGM control database; *n* > 800), AOH/LOH) all SVs detected in the cell lines were annotated. Unfiltered SVs for all lines are reported in Supplementary Tables 1–8. SVs detected with long-read data around the pathogenic variants in *SNCA* or *PRKN* are also reported in Supplementary Tables 9–13. An overview of the workflow is illustrated in Fig. 1.

### SNCA gene pathogenic variants (SFC831-03-05, SFC827-03-02)

**Fig. 2:**
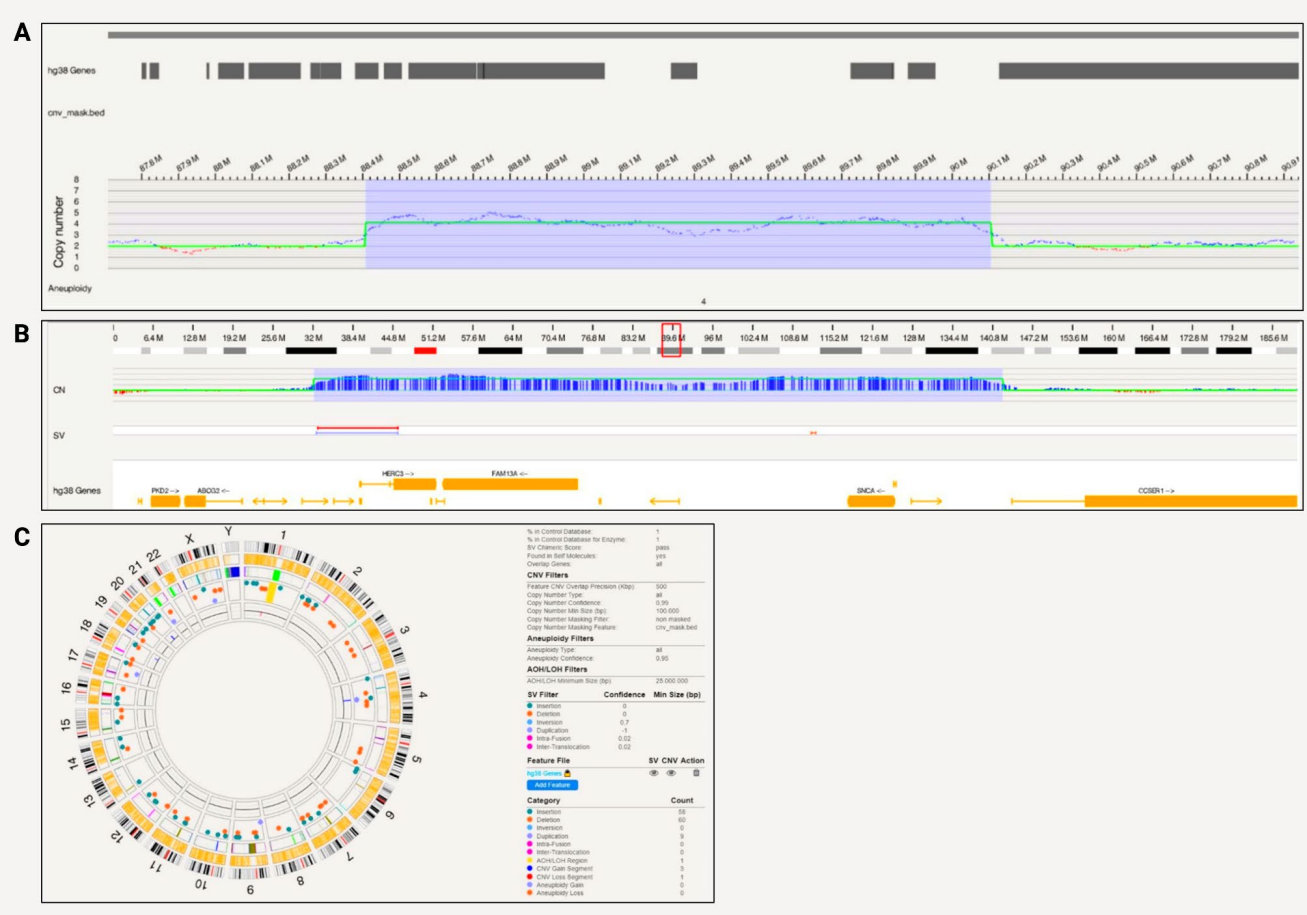
*SNCA* triplication detected with Bionano in a patient-derived iPSC line. **A**) A triplication spanning 1,696,488 bp that encompasses *SNCA*; **B**) Location of the triplication on chromosome 4 at 88,407,893 − 90,104,381 (hg38) that includes genes *HERC6*,* HERC5*,* PIGY*,* PYURF*,* PIGY-DT*,* HERC3*,* NAP1L5*,* FAM13A-AS1*,* FAM13A*,* TIGD2*,* GPRIN3*,* SNCA*,* SNCA-AS1*,* MMRN1*; **C**) Circos plot showing structural variants detected after filtering

Bionano optical mapping revealed a triplication in iPSC line SFC831-03-05 spanning 1,696,488 bp that encompasses *SNCA* (Fig. 2a). The triplication is on chromosome 4 at positions 88,407,893 − 90,104,381 (hg38) and includes genes *HERC6*,* HERC5*,* PIGY*,* PYURF*,* PIGY-DT*,* HERC3*,* NAP1L5*,* FAM13A-AS1*,* FAM13A*,* TIGD2*,* GPRIN3*,* SNCA*,* SNCA-AS1*,* MMRN1* (Fig. 2b). In terms of chromosomal abnormalities, no large inter- or intra-chromosomal translocations or gene fusions were detected, and no aneuploidy gain or loss found. Fifty-eight insertions and 60 deletions, one region of absence of heterozygosity, 9 duplications, 3 CNV gains and 1 loss were present and shown in the circos plot (Fig. 2c). Based on internally run Bionano samples to estimate the frequencies of these SVs, and annotation of pathogenicity with databases, no variants other than the triplication were considered pathogenic for PD.

From the long-read sequencing data, we obtained N50 = 9.8 kb and a mean base-calling phred-score of 16.3. From the Nanopore long-read sequencing data, Sniffles did not detect a triplication in the region of interest with a size of ~ 1.7 Mb. However, multiple CNVs with sizes of 37–149 Mb were detected that span the region of interest (Supplementary Table 9). Attempts to refine by adjusting the Sniffles parameters and utilizing the NGMLR alignment tool did not offer additional insights, and the exact triplication was not found. Still, a visible increase in the coverage was observed at the expected triplication when the alignment was visualized with the Integrative Genomics Viewer (Supplementary Fig. 2).

**Fig. 3:**
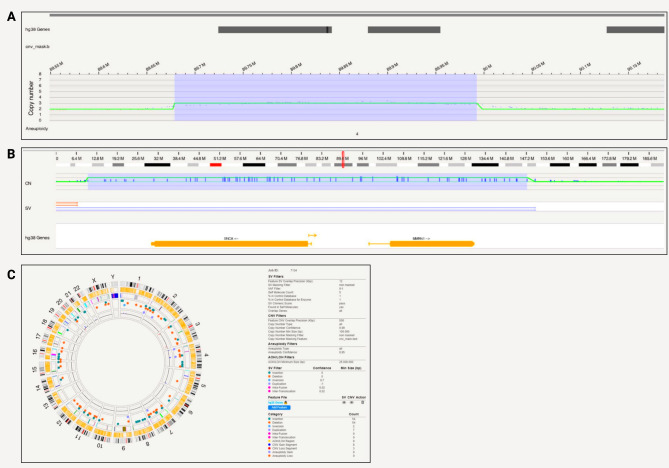
*SNCA* duplication detected with Bionano in a patient-derived iPSC line. **A**) A 313,859 bp long duplication that encompasses *SNCA*, spanning positions 89,678,642 − 89,992,501 on chromosome 4; **B**) Location of the triplication on chromosome 4 at 85388502–89998264 (hg38) that includes genes *SNCA*, *SNCA-AS1*, *MMRN1*; **C**) Circos plot showing structural variants detected after filtering

A 313,859 bp long duplication that encompasses *SNCA*, spanning positions 89,678,642 − 89,992,501 on chromosome 4 was detected in SFC827-03-02 with Bionano optical mapping (Fig. 3a). The region included genes *SNCA*,* SNCA-AS1*,* and MMRN1* (Fig. 3b). In terms of chromosomal abnormalities, no large inter or intra-chromosomal translocations or gene fusions were detected, and no aneuploidy gain or loss found. Fifty-four insertions and 54 deletions, 2 inversions, 7 duplications, 8 CNV gains and 3 losses were present and shown in the circos plot (Fig. 3c). Based on internally run Bionano samples to estimate the frequencies of these SVs, and annotation of pathogenicity with databases, no variants other than the duplication was considered pathogenic for PD.

From the long-read sequencing data, we obtained N50 = 18.67 kb and a mean base-calling phred-score of 12.2. Nanopore long-read sequencing did not detect a duplication of the size ~ 0.3 Mb in the region of interest with the Sniffles variant caller. However, when aligning only to the region of the expected duplication (chr4:87,678,642 − 91,992,501), other duplications with sizes of 0.6–2.7 Mb were detected that span the expected duplication (Supplementary Table 10). Similar to the triplication, adjusting the Sniffles parameter (i.e. coverage) and utilizing the NGMLR alignment tool did not offer additional insights, and the exact duplication was not found. Still, a visible increase in the coverage was observed at the expected duplication when the alignment was visualized with the Integrative Genomics Viewer (Supplementary Fig. 2).

### PRKN pathogenic variants (iPS-L-3034, iPS-L-3244 and iPS-L-10312)

**Fig. 4:**
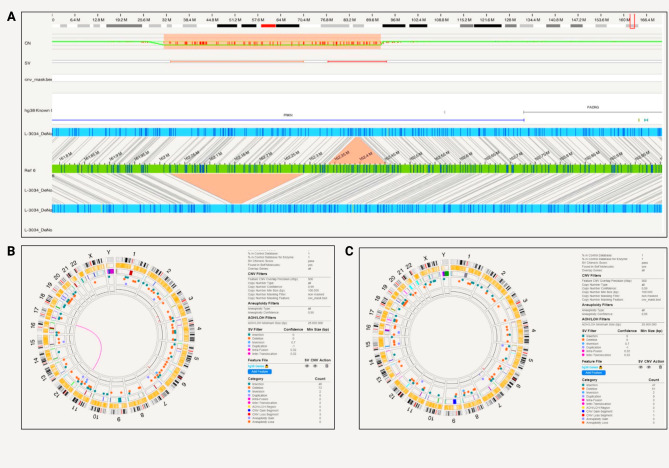
*PRKN* compound heterozygous variants detected with Bionano in iPSCs and the parental fibroblast. **A**) The compound heterozygous exon 2 and exon 3–5 deletion was captured with phase. The *PRKN* exon 2 deletion starts at position 162,338,358 and ends at 162,450,583, the *PRKN* exon 3–5 deletion starts at 162,029,361 and ends at 162,279,161 and partially overlaps with *PACRG*; **B**) Circos plot showing a translocation and other structural variants detected after filtering in the iPSC line from patient iPS-L-3034; **C**) Circos plot without the translocation present and structural variants detected after filtering in the parental fibroblast line L-3034

Within *PRKN*, a compound heterozygous exon 2 and exon 3–5 deletion was captured with phase on the Bionano for one patient iPSC cell line (iPS-L-3034) (Fig. 4a). The *PRKN* exon 2 deletion starts at position 162,338,358 and ends at 162,450,583, whereas the *PRKN* exon 3–5 deletion starts at 162,029,361 and ends at 162,279,161.

Multiplex-ligation-dependent probe amplification (MLPA) technique alone was unable to phase the deletions (data not shown). In terms of chromosomal abnormalities of the iPSCs, no gene fusions, no aneuploidy but two inter-chromosomal translocations were detected (Fig. 4b). The balanced translocations were not identified by routine single nucleotide variant karyotyping alone (data not shown). Forty-eight insertions and 73 deletions, 8 duplications, 2 inversions, 0 CNV gains and 3 losses were present and shown in the circos plot (Fig. 4b). In light of these findings that include a larger SV, we optically mapped the original fibroblast line, however, did not observe inter-chromosomal translocation in the line. There were no gene fusions and no aneuploidy, but we detected 47 insertions and 61 deletions, 9 duplications and 2 inversions, 1 CNV gain and 1 loss (Fig. 4c). In comparison to the iPSC line, different genetic variants were present in the fibroblasts (Supplementary Tables 3 and 6). In the unfiltered analysis, there were a total of 7596 SVs, 2617 deletions, 4648 insertions in the iPSC line compared to 7804 total SVs, 2673 deletions and 4746 insertions in the fibroblast lines. After filtering for a high-quality score (Q > 20) and rare variants (MAF < 0.01), there were no variants present in both lines.

To assess the quality of the two different biomaterials (iPSC and fibroblast) from the same patient (L-3034), we compared the molecule report for the Bionano run. The detailed molecule report showed a total number of 1,216,144 molecules for the iPSC line compared to 1,833,639 for the fibroblast culture and a total length of 312,589.23 Mb and 442,215.53 Mb, respectively. The average length of the iPSC line was 257.03 kb compared to 241.17 kb in the fibroblast culture. N50 of 239.63 kb for the iPSC and 237.37 kb for the fibroblast culture was achieved. Label density per 100 kb for the iPSC line was 16.84 resulting in a reference coverage of 101.22X compared to 14.55 per 100 kb for the fibroblast culture with a coverage for the reference of 143.19X.

From the long-read sequencing data, we obtained N50 = 12.55 kb and a mean base-calling phred-score of 15.7. Long-read sequencing was performed for the iPSC line, and the exon 2 deletion was confirmed. The sequencing revealed the deletion of exon 2 at position 162,336,451 − 162,448,855. Unfortunately, the exon 3–5 deletion was not found with long-reads when using the default Sniffles variant calling parameters. However, when adjusting parameters to counteract coverage changes within the large deletion, we detected both *PRKN* deletions, including the exon 3–5 deletion at position 162,029,842 − 162,280,393 (Supplementary Table 11). Additionally, the deletions were visible when assessing the alignment with the Integrative Genomics Viewer (Supplementary Fig. 3).

**Fig. 5:**
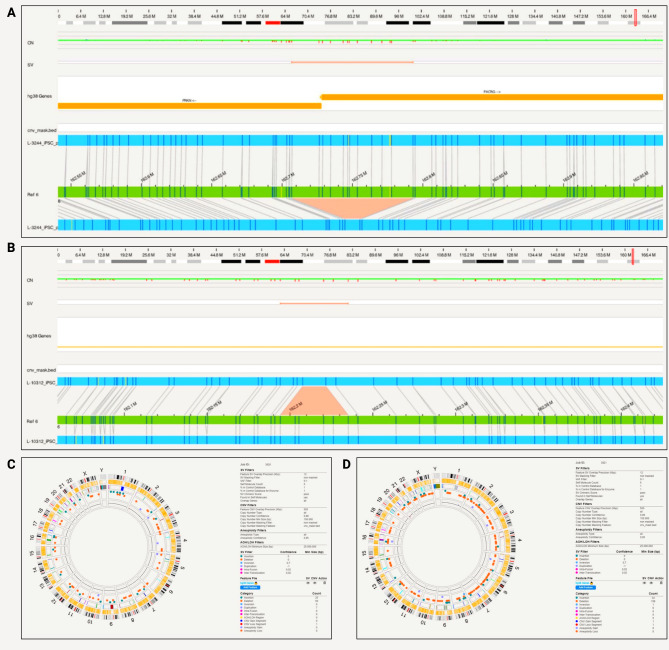
*PRKN* compound heterozygous variants detected with Bionano in iPSCs. **A**) A patient-derived iPSC line (iPS-L-3244) with *PRKN* exon 1 deletion. *PRKN* exon 1 deletion starts at position 162,716,506 and ends at 162,792,085 and partially overlaps with *PACRG*.; **B**) A patient-derived iPSC line (iPS-L-10312) with *PRKN* exon 4 deletion. *PRKN* exon 4 deletion starts at position 162,198,660 and ends at 162,225,645; **C**) Circos plot showing structural variants detected after filtering in iPS-L-3244; **D**) Circos plot with structural variants detected after filtering in iPS-L-10312

Furthermore, optical mapping detected *PRKN* deletions in exon 1 and exon 4 of the iPSC lines iPS-L-3244 and iPS-L-10312 (Fig. 5a and b). The *PRKN* exon 1 deletion starts at position 162,716,506 and ends at 162,792,085 and partially overlaps with *PACRG*. In this line, no large inter- or intra-chromosomal translocations or gene fusions were detected, and no aneuploidy gain or loss found. Twenty-five insertions and 59 deletions, 7 duplications, and one CNV loss were present and shown in a circos plot (Fig. 5c). From the long-read sequencing data, we obtained N50 = 22.58 kb and a mean base-calling phred-score = 12.2. Long-read sequencing data confirmed the *PRKN* exon 1 deletion at position 162,710,325 − 162,786,071 (Supplementary Table 12). Additionally, the deletion was visible when assessing the alignment with the Integrative Genomics Viewer (Supplementary Fig. 3).

In the third *PRKN*-mutant line (iPS-L-10312), the *PRKN* exon 4 deletion starts at position 162,198,660 and ends at 162,225,645. No large inter- or intra-chromosomal translocations or gene fusions were detected, and no aneuploidy gain or loss was found. We detected 34 insertions and 158 deletions, 1 inversion, 6 duplications, 1 CNV gain and 1 loss which are shown in the circos plot (Fig. 5d). From the long-read sequencing data, we obtained N50 = 13.41 kb and a mean base-calling phred-score of 16.0. Long-read sequencing data confirmed the *PRKN* exon 4 deletion (Supplementary Table 13). The deletion with a size of 27,092 bp was called with Sniffles and starts at position 162,200,764 and ends at 162,227,856. Additionally, the deletion was visible when assessing the alignment with the Integrative Genomics Viewer (Supplementary Fig. 3).

### Control iPSC lines (SFC065-03-03, SFC163-03-01)

To assess generally detected SVs in cell lines not related to PD, we performed OGM on two iPSC lines from healthy control individuals. In control line SFC065-03-03, no large inter or intra-chromosomal translocations or gene fusions were detected, and no aneuploidy gain or loss was found. Sixty-four insertions and 54 deletions, 3 inversions, 21 duplications, 10 CNV gain, and 1 loss were present. In the other control line, SFC163-03-01, no large inter or intra-chromosomal translocations or gene fusions were detected, and no aneuploidy gain or loss was found. We detected 59 insertions and 48 deletions, 2 inversions, 22 duplications, 1 CNV gain and 5 loss. These numbers were comparable to what we have observed in the patient iPSC lines (Supplementary Tables 7–8).

## Discussion

This is a proof of principle study showing that OGM can detect pathogenic SVs relevant to PD and the technique serves as a screening tool for iPSC’s genome integrity.

First, the Bionano OGM results include phasing and precision of the breakpoints of larger CNVs, solidifying the future use of this tool for gene discovery in PD. SVs in human diseases have been emerging, given the possibility of ascertaining these difficult genomic regions with newer technologies. For example, long-read sequencing revealed a GGC repeat expansion in ZFHX2 in SCA4 as disease-causing [22–25]. In monogenic PD, several genes have been identified to contain pathogenic CNVs in monogenic PD [26–29]. In this work, we present OGM results from cell lines and cultures with known pathogenic SVs in *SNCA* and *PRKN*. However, many identified GWAS risk loci lie within poorly annotated regions of the genome that contain repetitive sequences and transposable elements (TEs). In fact, the majority of risk variants lie in non-coding regions of the genome with no known functional consequence. This highlights the importance of investigating these regions for new pathogenic SVs. Enrichment of TEs has been seen at CNV breakpoints [30]. TEs such as Alu and LINE1 elements have been found around *SNCA* duplications and triplications [31]. Another gene (*FMR1*) with disease-associated REs on the X-chromosome may cause different phenotypes when mutated. Men with 55–200 CGG repeats may develop fragile X tremor/ataxia syndrome (FXTAS). Parkinsonism may form part of FXTAS, and the initial presentation may be L-dopa-responsive parkinsonism indistinguishable from PD [32]. *C9orf72* REs have been well characterized in the Amyotrophic lateral sclerosis / frontotemporal spectrum disorders [33]. In PD, *C9orf72* REs are relatively rare but account for some cases: a prevalence of 0.06% (*n* = 4/7,232) using a cutoff of > 60 repeat units as positive has been reported [34]. Another gene with pathogenic REs, *NOTCH2NLC*, was recently identified [35, 36] in Japanese patients with sporadic neuronal intranuclear inclusion disease [37]. A number of Chinese families with PD carried heterozygous GGC expansions larger than 65 (range 66–102) with possible anticipation [38]. Collectively, there is an indication of pathogenicity of SVs in PD and Parkinsonism-related disorders.

Our data show that OGM can detect large > 1 Mb triplications, duplications in *SNCA* and deletions in *PRKN*. In contrast, long-read sequencing did not detect all pathogenic variants. Comparing the number and length of the identified SVs between the different approaches (Supplementary Table 14), it becomes apparent that Oxford Nanopore Sequencing generally identified more SVs (up to 5141) compared to OGM (up to 240), but the OGM variant calls underwent a more stringent quality filtering. We infer that the results obtained from the Nanopore sequencing data might contain a higher number of common variants, smaller insertions/deletions undetected by OGM and/or artifacts.

With the long-read sequencing data and variant calling with Sniffles, it was possible to detect the *PRKN* deletions. However, Sniffles did not identify large triplications and duplications spanning the *SNCA* gene. We could detect larger CNVs spanning the *SNCA* gene; however, those varied in size and position compared to the variants detected with OGM. Still, the *SNCA* triplication and duplication were visible in the alignment, and an increased coverage was detected at the expected position of the SVs (Supplementary Fig. 4). Thus, it was potentially a limitation of the variant calling tool and not the long-read data itself. It has been previously demonstrated that the most utilized variant callers for long-read data have limitations when detecting large (i.e., > 50 kb) deletions and duplications [21]. Therefore, it might be helpful to complement the SV detection from long-read data with other approaches like *de novo* assembly or coverage quantification-based methods [21, 39]. Nevertheless, OGM would be a good tool to apply on families segregating with PD without a known genetic cause in combination with long-read sequencing.

Second, reprogramming fibroblasts and then differentiating iPSCs to other cell types allowed us to study novel candidate modifiers and disease mechanisms in these biologically relevant models [16, 40–43]. iPSC-derived neurons from *PRKN* mutation carriers (including iPS-L-3244) showed decreased complex I activity and altered mitochondrial network morphology [16]. iPSC lines with *SNCA* triplication (including SFC831-03-05) exhibited ~ 2.5-fold increase in endogenous α-synuclein monomer compared to healthy control neurons, which promoted α-synuclein seeding aggregation in the patient-derived neurons [41]. Other groups have also confirmed the utility of this model to increase our understanding of PD [44, 45]. However, due to the clonal expansion of one single cell, aberrant cytogenetic errors and somatic CNVs can arise from the reprogramming of somatic cells. As these changes could potentially affect cellular function or, in the case of genetic studies, the interpretation of inherited variants, genetic screening of iPSC is pertinent to perform. The gold standard practice to validate the genomic stability of iPSCs is G-band karyotype analysis. This technique is costly and requires the preparation and shipment of live cells. The resolution is limited to a chromosomal rearrangement of 5 Mb or larger [46]. We and others perform routine karyotyping of iPSCs by SNP microarray technology. This method is relatively inexpensive and has a better resolution (up to 100 kb) than karyotyping [47]. It detects CNV gain and loss and can be used to define the breakpoints and gene content of unbalanced chromosome abnormalities. It also provides information on ploidy levels and can detect copy number neutral loss of heterozygosity. However, this assay does not detect balanced rearrangements [48]. OGM is a capable tool to fully perform SV screening that not only includes karyotyping but even screening for more complex rearrangements and small SVs. Current cost of G-band karyotyping exceeds the cost of running a Bionano sample. Thus, a more comprehensive overview of genetic screening or genotyping iPSCs is possible at a lower cost. A recent study on CRISPR/Cas9 gene editing of iPSCs emphasized the necessity to examine cultures routinely for genomic alterations produced by gene editing strategies. They also analyzed the iPSCs by using OGM [49].

Limitations of OGM include the lack of base-pair resolution precision. Interestingly there were SV differences between fibroblast cultures and iPSC lines, which result most likely from the clonal selection and expansion during the reprogramming process. The run statistics from the Bionano molecules showed very comparable starting molecules and results. Thus, it would be important to include long-read sequencing data to confirm breakpoints or another method to confirm the SV of interest.

## Conclusions

In conclusion, OGM is a new method that can provide sufficient sensitivity and resolution for pathogenic SVs in PD. Future use of OGM in families affected with PD and an unknown genetic cause might help to elucidate novel variants implicated in disease pathogenesis. Furthermore, iPSC line screening by OGM encompasses karyotyping and can reveal genomic abnormalities at higher resolution.

## Electronic supplementary material

Below is the link to the electronic supplementary material.

Supplementary Material 1

Supplementary Material 2

Supplementary Material 3

## Data Availability

The datasets used and/or analyzed during the current study are available from the corresponding author on reasonable request.

## References

[1] Ho SS, Urban AE, Mills RE. Structural variation in the sequencing era. Nat Rev Genet. 2020;21(3):171–89. 31729472 10.1038/s41576-019-0180-9 PMC7402362

[2] Skrahina V, Gaber H, Vollstedt EJ, Forster TM, Usnich T, Curado F, et al. The Rostock International Parkinson’s Disease (ROPAD) study: protocol and initial findings. Mov Disord. 2021;36(4):1005–10. 33314351 10.1002/mds.28416 PMC8246975

[3] Nalls MA, Blauwendraat C, Vallerga CL, Heilbron K, Bandres-Ciga S, Chang D, et al. Identification of novel risk loci, causal insights, and heritable risk for Parkinson’s disease: a meta-analysis of genome-wide association studies. Lancet Neurol. 2019;18(12):1091–102. 31701892 10.1016/S1474-4422(19)30320-5 PMC8422160

[4] Farrer M, Maraganore DM, Lockhart P, Singleton A, Lesnick TG, de Andrade M, et al. Alpha-synuclein gene haplotypes are associated with Parkinson’s disease. Hum Mol Genet. 2001;10(17):1847–51. 11532993 10.1093/hmg/10.17.1847

[5] Singleton AB, Farrer M, Johnson J, Singleton A, Hague S, Kachergus J, et al. Alpha-synuclein locus triplication causes Parkinson’s disease. Science. 2003;302(5646):841. 14593171 10.1126/science.1090278

[6] Djarmati A, Hedrich K, Svetel M, Schafer N, Juric V, Vukosavic S, et al. Detection of Parkin (PARK2) and DJ1 (PARK7) mutations in early-onset Parkinson disease: parkin mutation frequency depends on ethnic origin of patients. Hum Mutat. 2004;23(5):525. 15108293 10.1002/humu.9240

[7] Klein C, Lohmann-Hedrich K, Rogaeva E, Schlossmacher MG, Lang AE. Deciphering the role of heterozygous mutations in genes associated with parkinsonism. Lancet Neurol. 2007;6(7):652–62. 17582365 10.1016/S1474-4422(07)70174-6

[8] Klein C, Pramstaller PP, Kis B, Page CC, Kann M, Leung J, et al. Parkin deletions in a family with adult-onset, tremor-dominant parkinsonism: expanding the phenotype. Ann Neurol. 2000;48(1):65–71. 10894217

[9] Assou S, Bouckenheimer J, De Vos J. Concise Review: assessing the Genome Integrity of Human Induced Pluripotent Stem cells: what Quality Control Metrics? Stem Cells. 2018;36(6):814–21. 29441649 10.1002/stem.2797

[10] Barseghyan H, Tang W, Wang RT, Almalvez M, Segura E, Bramble MS, et al. Next-generation mapping: a novel approach for detection of pathogenic structural variants with a potential utility in clinical diagnosis. Genome Med. 2017;9(1):90. 29070057 10.1186/s13073-017-0479-0 PMC5655859

[11] Neveling K, Mantere T, Vermeulen S, Oorsprong M, van Beek R, Kater-Baats E, et al. Next-generation cytogenetics: Comprehensive assessment of 52 hematological malignancy genomes by optical genome mapping. Am J Hum Genet. 2021;108(8):1423–35. 34237281 10.1016/j.ajhg.2021.06.001 PMC8387283

[12] Dremsek P, Schwarz T, Weil B, Malashka A, Laccone F, Neesen J. Optical genome mapping in Routine Human Genetic Diagnostics-its advantages and limitations. Genes (Basel). 2021;12(12):1958. 34946907 10.3390/genes12121958 PMC8701374

[13] Midha MK, Wu M, Chiu KP. Long-read sequencing in deciphering human genetics to a greater depth. Hum Genet. 2019;138(11–12):1201–15. 31538236 10.1007/s00439-019-02064-y

[14] van Dijk EL, Jaszczyszyn Y, Naquin D, Thermes C. The third revolution in sequencing technology. Trends Genet. 2018;34(9):666–81. 29941292 10.1016/j.tig.2018.05.008

[15] Haenseler W, Zambon F, Lee H, Vowles J, Rinaldi F, Duggal G, et al. Excess alpha-synuclein compromises phagocytosis in iPSC-derived macrophages. Sci Rep. 2017;7(1):9003. 28827786 10.1038/s41598-017-09362-3 PMC5567139

[16] Zanon A, Kalvakuri S, Rakovic A, Foco L, Guida M, Schwienbacher C, et al. SLP-2 interacts with Parkin in mitochondria and prevents mitochondrial dysfunction in parkin-deficient human iPSC-derived neurons and Drosophila. Hum Mol Genet. 2017;26(13):2412–25. 28379402 10.1093/hmg/ddx132 PMC6192413

[17] Baumann H, Jahn M, Muenchau A, Trilck-Winkler M, Lohmann K, Seibler P. Generation and characterization of eight human-derived iPSC lines from affected and unaffected THAP1 mutation carriers. Stem Cell Res. 2018;33:60–4. 30316041 10.1016/j.scr.2018.09.018

[18] Li H. Minimap2: pairwise alignment for nucleotide sequences. Bioinformatics. 2018;34(18):3094–100. 29750242 10.1093/bioinformatics/bty191 PMC6137996

[19] Li H, Handsaker B, Wysoker A, Fennell T, Ruan J, Homer N, et al. The sequence Alignment/Map format and SAMtools. Bioinformatics. 2009;25(16):2078–9. 19505943 10.1093/bioinformatics/btp352 PMC2723002

[20] Zhou A, Lin T, Xing J. Evaluating nanopore sequencing data processing pipelines for structural variation identification. Genome Biol. 2019;20(1):237. 31727126 10.1186/s13059-019-1858-1 PMC6857234

[21] Cuenca-Guardiola J, de la Morena-Barrio B, Garcia JL, Sanchis-Juan A, Corral J, Fernandez-Breis JT. Improvement of large copy number variant detection by whole genome nanopore sequencing. J Adv Res. 2023;50:145–58. 36323370 10.1016/j.jare.2022.10.012 PMC10403694

[22] Wallenius J, Kafantari E, Jhaveri E, Gorcenco S, Ameur A, Karremo C, et al. Exonic trinucleotide repeat expansions in ZFHX3 cause spinocerebellar ataxia type 4: a poly-glycine disease. Am J Hum Genet. 2024;111(1):82–95. 38035881 10.1016/j.ajhg.2023.11.008 PMC10806739

[23] Su S, Cui MY, Gui Z, Guo QQ, Ren H, Ma SF, et al. First detection of Candidatus Rickettsia tarasevichiae in Hyalomma marginatum ticks. PLoS ONE. 2024;19(2):e0296757. 38306367 10.1371/journal.pone.0296757 PMC10836667

[24] Rudaks LI, Yeow D, Kumar KR. SCA4 unravelled after more than 25 years using advanced genomic technologies. Mov Disord. 2024;39(3):457–61. 38525586 10.1002/mds.29738

[25] Chen Z, Gustavsson EK, Macpherson H, Anderson C, Clarkson C, Rocca C, et al. Adaptive long-read sequencing reveals GGC repeat expansion in ZFHX3 Associated with Spinocerebellar Ataxia Type 4. Mov Disord. 2024;39(3):486–97. 38197134 10.1002/mds.29704

[26] Pankratz N, Dumitriu A, Hetrick KN, Sun M, Latourelle JC, Wilk JB, et al. Copy number variation in familial Parkinson disease. PLoS ONE. 2011;6(8):e20988. 21829596 10.1371/journal.pone.0020988 PMC3149037

[27] Wang L, Nuytemans K, Bademci G, Jauregui C, Martin ER, Scott WK, et al. High-resolution survey in familial Parkinson disease genes reveals multiple independent copy number variation events in PARK2. Hum Mutat. 2013;34(8):1071–4. 23616242 10.1002/humu.22344 PMC4464794

[28] Kay DM, Stevens CF, Hamza TH, Montimurro JS, Zabetian CP, Factor SA, et al. A comprehensive analysis of deletions, multiplications, and copy number variations in PARK2. Neurology. 2010;75(13):1189–94. 20876472 10.1212/WNL.0b013e3181f4d832 PMC3013490

[29] Book A, Guella I, Candido T, Brice A, Hattori N, Jeon B, et al. A Meta-analysis of alpha-synuclein multiplication in familial parkinsonism. Front Neurol. 2018;9:1021. 30619023 10.3389/fneur.2018.01021 PMC6297377

[30] Bose P, Hermetz KE, Conneely KN, Rudd MK. Tandem repeats and G-rich sequences are enriched at human CNV breakpoints. PLoS ONE. 2014;9(7):e101607. 24983241 10.1371/journal.pone.0101607 PMC4090240

[31] Ross OA, Braithwaite AT, Skipper LM, Kachergus J, Hulihan MM, Middleton FA, et al. Genomic investigation of alpha-synuclein multiplication and parkinsonism. Ann Neurol. 2008;63(6):743–50. 18571778 10.1002/ana.21380 PMC3850281

[32] Hall DA, Jennings D, Seibyl J, Tassone F, Marek K. FMR1 gene expansion and scans without evidence of dopaminergic deficits in parkinsonism patients. Parkinsonism Relat Disord. 2010;16(9):608–11. 20702130 10.1016/j.parkreldis.2010.07.006 PMC2963704

[33] DeJesus-Hernandez M, Mackenzie IR, Boeve BF, Boxer AL, Baker M, Rutherford NJ, et al. Expanded GGGGCC hexanucleotide repeat in noncoding region of C9ORF72 causes chromosome 9p-linked FTD and ALS. Neuron. 2011;72(2):245–56. 21944778 10.1016/j.neuron.2011.09.011 PMC3202986

[34] Theuns J, Verstraeten A, Sleegers K, Wauters E, Gijselinck I, Smolders S, et al. Global investigation and meta-analysis of the C9orf72 (G4C2)n repeat in Parkinson disease. Neurology. 2014;83(21):1906–13. 25326098 10.1212/WNL.0000000000001012 PMC4248456

[35] Fiddes IT, Lodewijk GA, Mooring M, Bosworth CM, Ewing AD, Mantalas GL, et al. Human-specific NOTCH2NL genes affect Notch Signaling and cortical neurogenesis. Cell. 2018;173(6):1356–e6922. 29856954 10.1016/j.cell.2018.03.051 PMC5986104

[36] Suzuki IK, Gacquer D, Van Heurck R, Kumar D, Wojno M, Bilheu A, et al. Human-specific NOTCH2NL genes expand cortical neurogenesis through Delta/Notch Regulation. Cell. 2018;173(6):1370–e8416. 29856955 10.1016/j.cell.2018.03.067 PMC6092419

[37] Sone J, Mitsuhashi S, Fujita A, Mizuguchi T, Hamanaka K, Mori K, et al. Long-read sequencing identifies GGC repeat expansions in NOTCH2NLC associated with neuronal intranuclear inclusion disease. Nat Genet. 2019;51(8):1215–21. 31332381 10.1038/s41588-019-0459-y

[38] Deng J, Gu M, Miao Y, Yao S, Zhu M, Fang P, et al. Long-read sequencing identified repeat expansions in the 5’UTR of the NOTCH2NLC gene from Chinese patients with neuronal intranuclear inclusion disease. J Med Genet. 2019;56(11):758–64. 31413119 10.1136/jmedgenet-2019-106268

[39] Shafin K, Pesout T, Lorig-Roach R, Haukness M, Olsen HE, Bosworth C, et al. Nanopore sequencing and the Shasta toolkit enable efficient de novo assembly of eleven human genomes. Nat Biotechnol. 2020;38(9):1044–53. 32686750 10.1038/s41587-020-0503-6 PMC7483855

[40] Seibler P, Burbulla LF, Dulovic M, Zittel S, Heine J, Schmidt T, et al. Iron overload is accompanied by mitochondrial and lysosomal dysfunction in WDR45 mutant cells. Brain. 2018;141(10):3052–64. 30169597 10.1093/brain/awy230 PMC7190033

[41] Tanudjojo B, Shaikh SS, Fenyi A, Bousset L, Agarwal D, Marsh J, et al. Phenotypic manifestation of alpha-synuclein strains derived from Parkinson’s disease and multiple system atrophy in human dopaminergic neurons. Nat Commun. 2021;12(1):3817. 34155194 10.1038/s41467-021-23682-z PMC8217249

[42] Bogetofte H, Ryan BJ, Jensen P, Schmidt SI, Vergoossen DLE, Barnkob MB, et al. Post-translational proteomics platform identifies neurite outgrowth impairments in Parkinson’s disease GBA-N370S dopamine neurons. Cell Rep. 2023;42(3):112180. 36870058 10.1016/j.celrep.2023.112180 PMC7617855

[43] Knappe E, Rudolph F, Klein C, Seibler P. Cytokine profiling in human iPSC-Derived dopaminergic neuronal and microglial cultures. Cells. 2023;12(21). 10.3390/cells12212535 PMC10650774 37947613

[44] Kim JW, Yin X, Jhaldiyal A, Khan MR, Martin I, Xie Z, et al. Defects in mRNA translation in LRRK2-Mutant hiPSC-Derived dopaminergic neurons lead to Dysregulated Calcium Homeostasis. Cell Stem Cell. 2020;27(4):633–45. e7. 32846140 10.1016/j.stem.2020.08.002 PMC7542555

[45] Burbulla LF, Song P, Mazzulli JR, Zampese E, Wong YC, Jeon S, et al. Dopamine oxidation mediates mitochondrial and lysosomal dysfunction in Parkinson’s disease. Science. 2017;357(6357):1255–61. 28882997 10.1126/science.aam9080 PMC6021018

[46] Elliott AM, Elliott KA, Kammesheidt A. High resolution array-CGH characterization of human stem cells using a stem cell focused microarray. Mol Biotechnol. 2010;46(3):234–42. 20524159 10.1007/s12033-010-9294-1

[47] D’Antonio M, Woodruff G, Nathanson JL, D’Antonio-Chronowska A, Arias A, Matsui H, et al. High-throughput and cost-effective characterization of Induced Pluripotent Stem cells. Stem Cell Rep. 2017;8(4):1101–11. 10.1016/j.stemcr.2017.03.011 PMC5390243 28410643

[48] Shah MS, Cinnioglu C, Maisenbacher M, Comstock I, Kort J, Lathi RB. Comparison of cytogenetics and molecular karyotyping for chromosome testing of miscarriage specimens. Fertil Steril. 2017;107(4):1028–33. 28283267 10.1016/j.fertnstert.2017.01.022

[49] Gallego Villarejo L, Gerding WM, Bachmann L, Hardt LHI, Bormann S, Nguyen HP, Müller T. Optical genome mapping reveals genomic alterations upon gene editing in hiPSCs: implications for neural tissue differentiation and brain organoid research. Cells. 2024;13(6):507. 10.3390/cells13060507. 10.3390/cells13060507 PMC10969360 38534351

