# Supplementary material for: Optical genome mapping of structural variants in Parkinson’s disease-related induced pluripotent stem cells
Source: BMC Genomics. 2024 Oct 19;25:980. doi: 10.1186/s12864-024-10902-1 (PMC11490025; doi:10.1186/s12864-024-10902-1)
Supplement: Supplementary file 1 — Supplementary Material 1 [file 12864_2024_10902_MOESM1_ESM.pdf]

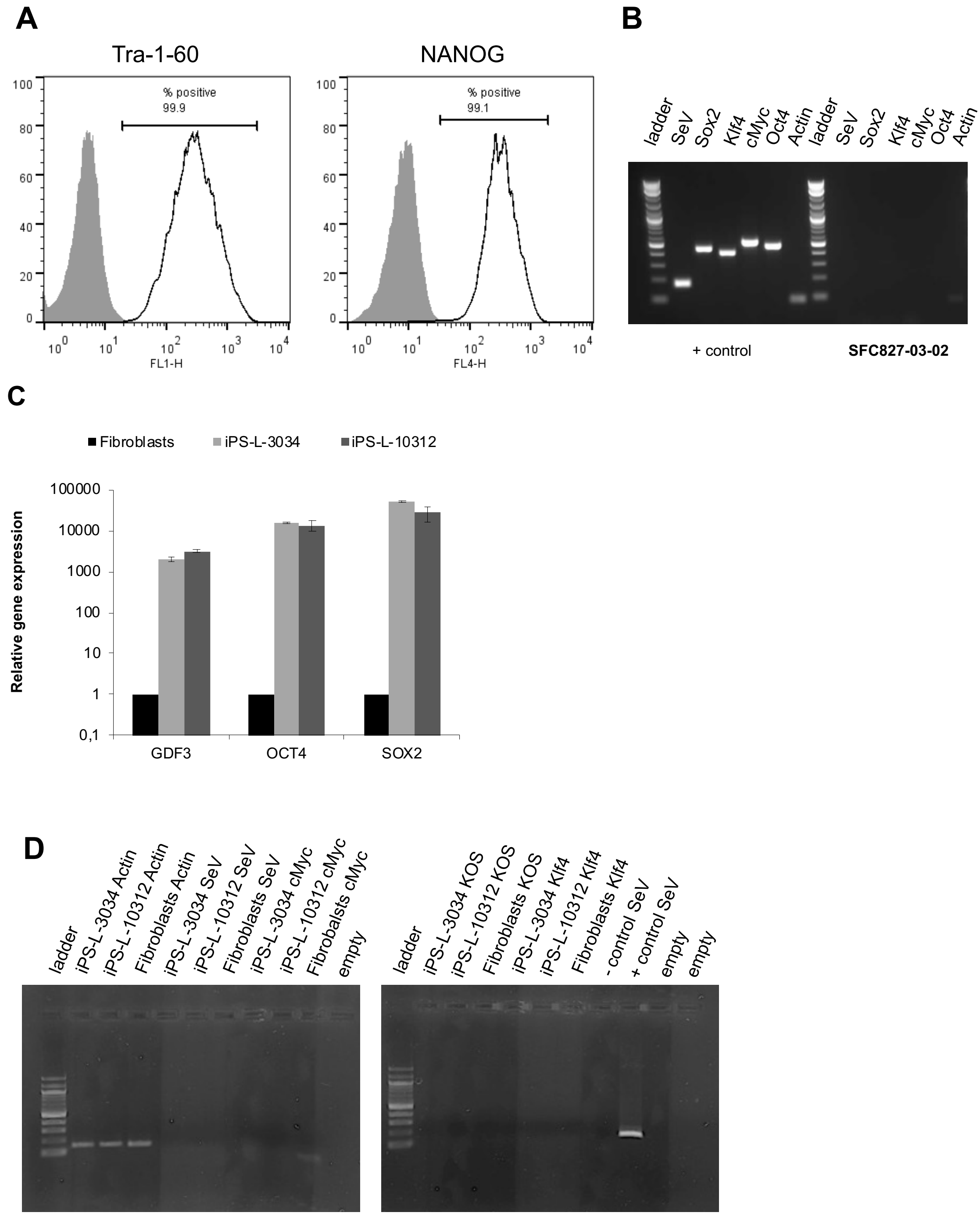

Supplementary Figure 1. Characterization of previously unpublished iPSC lines. A-B) SFC827-03-02; A) FACS analysis of pluripotency markers Tra-1-60 and NANOG for iPSC line SFC827-03-02. B) Absence of Sendai virus components as demonstrated after RT-PCR amplification (SeV, Sendai virus). Positive control (fibroblasts infected 5 days previously) was run in parallel. C-D) iPS-L-3034, iPS-L-10312; C) RT-PCR assessment of pluripotency markers GDF3, OCT4, and SOX2 in iPSC lines normalized to beta-actin and relative to fibroblasts. D) Absence of Sendai virus components as demonstrated after RT-PCR amplification. Positive control (fibroblasts infected 5 days previously) was run in parallel.

SFC831-03-05: SNCA triplication (1.6Mb)

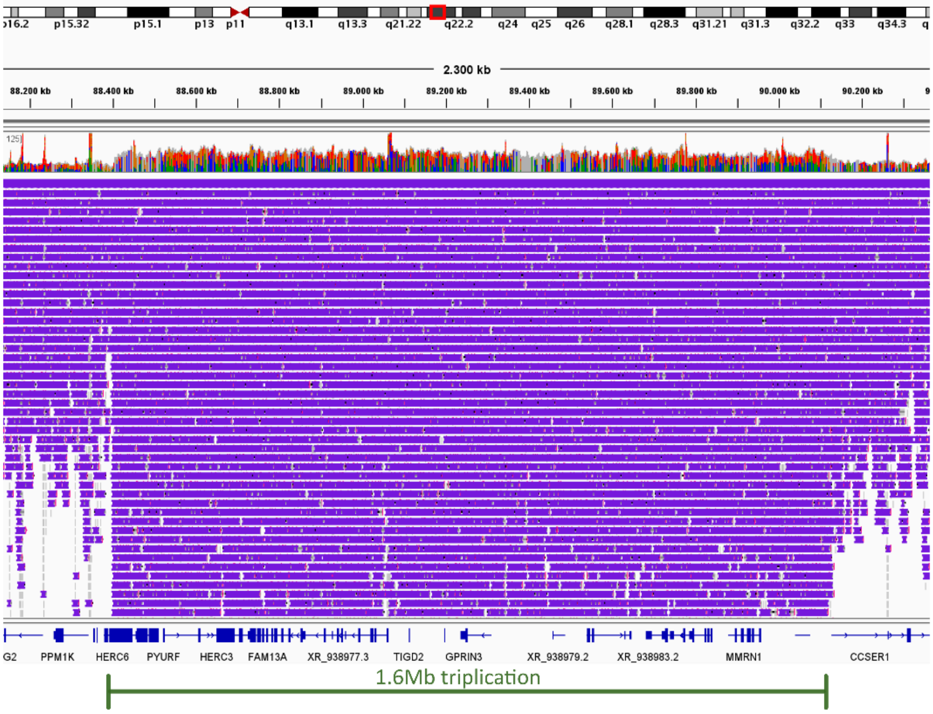

SFC827-03-02: SNCA duplication (0.3Mb)

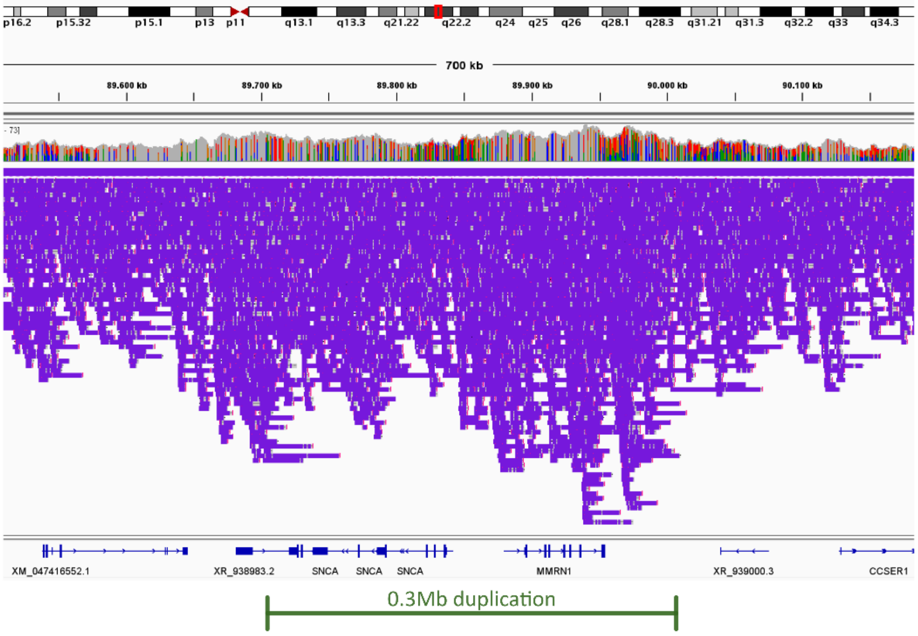

Supplementary Figure 2. The plots show the alignment of the long-read sequencing data around the genomic position where optical genome mapping detected the triplication or duplication copy number variant. The software Integrative Genomics Viewer was used, and the alignment was performed with NGMLR.

iPS-L-3034: compound heterozygous PRKN exon3-5 (0.25Mb) & exon 2 (0.1Mb) deletion

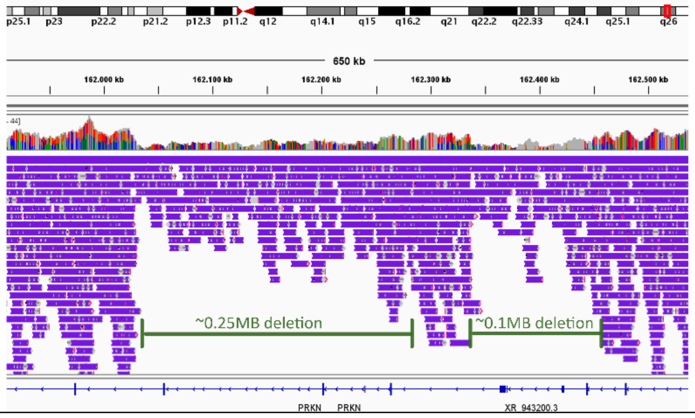

iPS-L-3244: heterozygous PRKN exon1 deletion (0.08Mb)

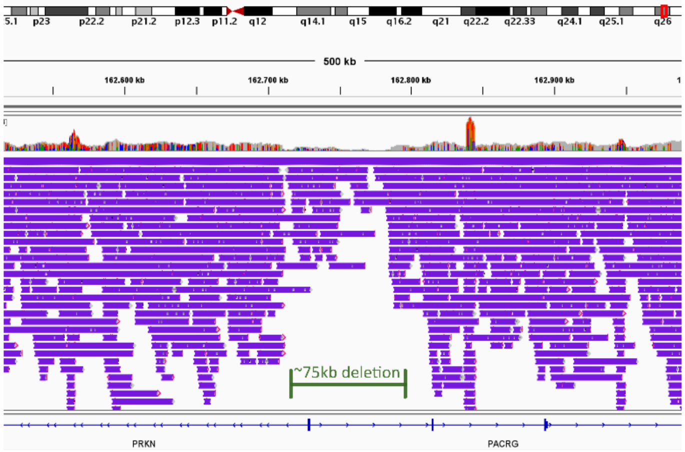

iPS-L-10312: heterozygous PRKN exon4 deletion (0.03Mb)

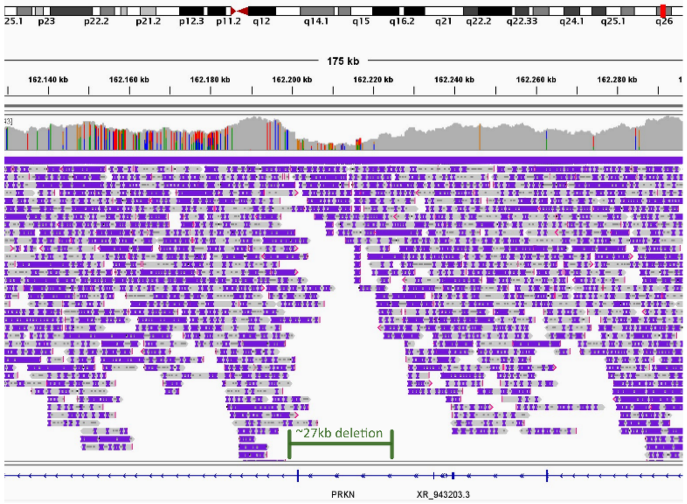

Supplementary Figure 3. The plots show the alignment of the long-read sequencing data around the genomic position where the deletions within the *PRKN* gene were detected from the long-read data. The software Integrative Genomics Viewer was used, and the alignment was performed with minimap2.

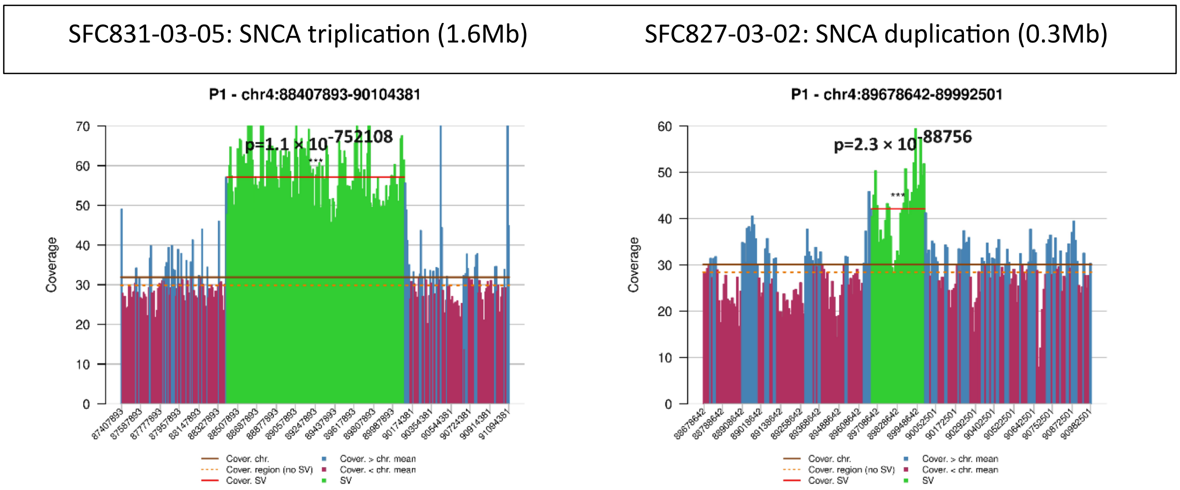

Supplementary Figure 4. The difference between the coverage of the triplication or duplication and the surrounding area. The plots were generated with the disCoverage software tool. The statistical comparison between the coverage was performed by disCoverage as well.
