# Supplementary material for: Optical genome mapping of structural variants in Parkinson’s disease-related induced pluripotent stem cells
Source: BMC Genomics. 2024 Oct 19;25:980. doi: 10.1186/s12864-024-10902-1 (PMC11490025; doi:10.1186/s12864-024-10902-1)
Supplement: Supplementary file 2 — Supplementary Material 2 [file 12864_2024_10902_MOESM2_ESM.pdf]

Supplementary Table 14. Optical genome mapping and Nanopore long-read sequencing were compared by the number and length of identified structural variants (SV). Unless otherwise indicated, the DNA was derived from iPSC lines.

N of INS	Mean INS length (± SD)	N of DEL	Mean DEL length (± SD)	N of INV	Mean INV length (± SD)	N of DUP	Mean DUP length (± SD)	total SV
**SFC831-03-05, Optical genome mapping**								
139	3227 (±6160)	91	5546 (±13550)	0	NA	4	555300 (±780273)	234
**SFC831-03-05, Nanopore Sequencing**								
3008	2000 (±2721)	2066	653855 (±8342987)	50	31532040 (±36977885)	17	23533631 (±42522638)	5141
**SFC827-03-02, Optical genome mapping**								
128	4970 (±12945)	90	50051 (±421729)	0	NA	5	1143750 (±1937702)	223
**SFC827-03-02, Nanopore Sequencing**								
997	7393 (±5906)	65	31148 (±249497)	6	1968511 (±1547190)	8	1309265 (±1049652)	1076
**iPS-L-3034, Optical genome mapping**								
147	2604 (±3829)	80	17342 (±67765)	1	15530	1	192345	229
**L-3034 (Fibroblast line), Optical genome mapping**								
156	3069 (±7912)	76	15415 (±64830)	0	NA	0	NA	232
**iPS-L-3034, Nanopore Sequencing**								
1796	1461 (±2536)	1565	466028 (±6086730)	26	37004333 (±48137264)	3	9745447 (±16862528)	3390
**iPS-L-3244, Optical genome mapping**								
149	3563 (±9415)	91	5937 (±11515)	0	NA	0	NA	240
**iPS-L-3244, Nanopore Sequencing**								
2610	2855 (±4597)	1780	94198 (±2820108)	15	35708685 (±51677059)	3	16188231 (±28020535)	4408
**iPS-L-10312, Optical genome mapping**								
143	4017 (±11004)	95	5745 (±12074)	0	NA	0	NA	238
**iPS-L-10312, Nanopore Sequencing**								
3063	2772 (±3355)	1852	368102 (±5018483)	46	40761950 (±46692210)	13	20188411 (±30043006)	4974

N = Number of individual SV types; INS = Insertion; DEL = Deletion; INV = Inversion; DUP = Duplication
The mean length of the SV types and the corresponding SD are displayed in bp.
